# Preserving Microstructure Enhances Cohesion and Mechanical Performance in *Spirulina*-Based 3D-Printed Biomaterials

**DOI:** 10.1021/acsaenm.5c01105

**Published:** 2026-01-16

**Authors:** Amelia Burns, Israel Kellersztein, Chiara Daraio

**Affiliations:** † Division of Biology and Biological Engineering, 6469 California Institute of Technology, 1200 E. California Blvd., Pasadena, California 91125, United States; ‡ Division of Engineering and Applied Science, 6469 California Institute of Technology, 1200 E. California Blvd., Pasadena, California 91125, United States

**Keywords:** 3D printing, *Spirulina*, microalgae, biocomposites, processing, sustainable materials, microstructure

## Abstract

*Spirulina platensis* is a promising bioresource for developing structural materials, offering a renewable alternative to conventional polymers due to its rapid growth and characteristic helical microstructure. While its biochemical properties have been widely studied, the role of cellular morphology in determining macroscale mechanical performance remains underexplored. In this work, we examine how maintaining versus disrupting *Spirulina*’s native trichome structure and cell walls impacts the cohesion, rheology, and mechanical behavior of 3D-printed biomaterials. Using hydroxyethyl cellulose (HEC) as a binder, we developed two classes of bioinks: trichome biocomposites, based on freeze-dried *Spirulina* trichomes, and lysed biocomposites, formed from thermally lysed *Spirulina* cells. Differential scanning calorimetry revealed stronger molecular interactions between lysed cells and HEC, while trichomes contributed instead via physical interlocking and structural integrity of the cell wall. Despite weaker molecular interactions, trichome-based biocomposite bioinks exhibited higher viscosity, improved printability, and higher rheological yield stress by up to 499%. Upon dehydration, trichome biocomposites showed lower shrinkage and higher mechanical performance under compression, with normalized compressive modulus and yield strength significantly exceeding that of lysed biocomposites (by up to 107% and 108%, respectively). These effects are attributed to mechanical interlocking and enhanced stress transfer through intact cell walls. Our findings demonstrate that preserving biological microstructure may enable improved material cohesion and function, offering design principles for scalable, sustainable biofabrication of algae-based structural materials.

## Graphical abstract

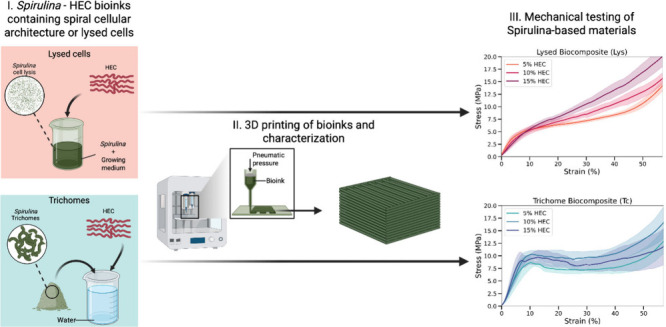

## 1 Introduction

The continuous production, use, and disposal of petroleum-based plastics such as polyethylene and polypropylene have significantly contributed to waste pollution,^1^ creating a demand for sustainable, biodegradable alternatives for applications across multiple industries including packaging, consumer products, and construction.^2^ Bioplastics derived from crops such as corn and potatoes (e.g., polylactic acid (PLA) and starch blends) have been developed, but lack long-term sustainability due to high production costs and the fact that their production competes with the demand for these crops as food sources.^2–4^ Other biocomposite materials composed of lignocellulosic fibers also pose environmental and economic concerns due to their energy-intensive processing.^5^ While renewable, wood contributes to deforestation and often fails to match the functionality of plastics.^6,7^

Microalgae have been studied in recent years for its significant potential as a valuable biomass resource for use in more cost-effective and sustainable biomaterials.^8–10^ These photosynthetic unicellular organisms exhibit a diverse biochemical composition rich in proteins, lipids, and polysaccharides, and thrive in in a variety of conditions, including marine and freshwater environments as well as controlled bioreactors without competing for traditional agricultural resources.^11,12^ As photoautotrophs, they also aid in carbon sequestration by converting carbon dioxide into oxygen.^13–15^

Microalgae have been incorporated into various structural materials, including polymer blends and composites, fabricated using conventional fabrication methods such as compression and injection molding.^16–18^ However, these methods require high temperatures and thus increased energy consumption. In contrast, 3D printing offers a more sustainable manufacturing approach by eliminating the need for excessive heat and energy-intensive processing, and enables greater control and flexibility over material properties and design.^19–21^

Recent studies have explored the formulation of algae-based biocomposites for extrusion-based 3D printing, using dried and powdered microalgae cells mixed with water to form bioinks printable at room temperature.^21–23^ For instance, it was found that the addition of cellulose fibers to *Spirulina*-based bioinks improve their mechanical strength, with 20 wt % cellulose fibers increasing compressive strength to 16.4 MPa.^22^ Similarly, another study showed that the addition of hydroxyethyl cellulose (HEC) enhanced the structural integrity of bioinks composed of the microalgae species *Chlorella vulgaris*. Fourier transform infrared spectroscopy (FTIR) analysis revealed that the hydrogen bonding between HEC molecules and the microalgae cells plays a crucial role in reinforcing these materials, contributing to improved compression and bending moduli (566 and 1630 MPa, respectively).^21^

*Spirulina* possesses a unique microstructure, consisting of individual cells stacked into multicellular helical trichomes than can physically entangle at the bulk level.^24,25^ However, this architecture is susceptible to disruption by processing methods such as sonication, heating, and chemical treatments, which can fragment the trichomes and release intracellular components.^26,27^ While it is well-established that cellular microstructure influences the mechanical behavior of biological materials,^28–31^ the specific role of *Spirulina*’s architecture on the performance of *Spirulina*-based materials remains largely unexplored. A study found that trichome fragmentation via sonication improved tensile strength in *Spirulina*-PLA biocomposites by up to 25%,^32^ but did not isolate the structural function of *Spirulina* itself.

In this study, we investigate how *Spirulina* microstructure and hydroxyethyl cellulose (HEC) concentration influence the cohesion and mechanical behavior of algae-based materials. Two forms of *Spirulina* were compared: trichomes maintained through freeze-drying, and lysed cells obtained by thermal treatment. These were combined with varying amounts of HEC to create formulations suitable for extrusion-based 3D printing. We define a trichome biocomposite (abbreviated as Tc) as a system in which trichomes act as structural reinforcements within an HEC matrix, while lysed biocomposites (abbreviated as Ly) refer to mixtures of lysed cells and HEC with no distinct reinforcing. Thermal analysis revealed stronger mixing between lysed biomass and HEC, while composites retained more distinct thermal transitions, suggesting limited molecular integration and dominant physical cohesion. However, rheology and mechanical testing showed that HEC also contributes to the strength of biocomposites, indicating some level of interaction with intact trichomes. Overall, trichome biocomposites outperformed lysed biocomposites in yield stress and stiffness, highlighting the importance of cellular architecture in governing material performance. These findings offer practical guidelines for tuning algae-based materials through biomass processing and binder selection, with relevance for sustainable packaging, structural panels, and future biofabrication strategies.

## 2 Methods

### 2.1 Materials

All materials were used as received unless otherwise specified. *Spirulina platensis* was purchased from Oasia Farms (Little Rock, California). The *Spirulina* was cultivated in an open pond, and excess of growth medium was removed using a mechanical press prior to freezing and storage. All *Spirulina* used in sample fabrication was sourced from the same crop cultivated at the same time, ensuring comparable growth conditions across all samples. 2-hydroexyethyl cellulose (HEC, M_v_ ∼ 1,300,000) was purchased from Sigma-Aldrich (MKE, USA). Osmium tetroxide (OsO_4_) 4% aqueous solution was obtained from Electron Microscopy Sciences (PA, USA).

### 2.2 Confocal Microscopy

Confocal imaging was used to evaluate the effect of freeze-drying and thermal treatment on *Spirulina* cell morphology. Untreated *Spirulina* biomass was imaged as reference. A drop of each biomass suspension in deionized water was placed on a glass slide and covered with a thin layer of agar to immobilize the sample during imaging.

Maximum intensity projection images were taken from z-stacks acquired using an Andor Dragonfly 202 spinning disk system mounted on a Nikon Ti2-E inverted microscope, equipped with a 4.2-megapixel Zyla sCMOS camera. A Nikon Lambda D 60x oil immersion objective (NA 1.42, working distance 0.15 mm) was used for image acquisition. Excitation was provided by a 640 nm laser, and fluorescence emission was collected using a 698/77 nm bandpass filter. Image processing was performed using ImageJ software.

### 2.3 Thermogravimetric Analysis (TGA)

The initial water content of the *Spirulina* was measured using thermogravimetric analysis (TGA). Measurements were performed on a Discovery TGA 550 (TA Instruments, USA) under a nitrogen atmosphere, using a temperature range of 25–250 °C and a heating rate of 10 °C/min. Water content was calculated as the percentage of mass lost by 110 °C (Figure S1).

### 2.4 ATR-FTIR Spectroscopy

Attenuated total reflectance-infrared (ATR-FIR) spectroscopy was used to analyze the biochemical composition of *Spirulina* samples and to assess cohesive interactions with HEC. Spectra were collected using a Nicolet 6700 FTIR spectrometer (Thermo Scientific) equipped with an ATR accessory. Measurements were performed over the range of 400–4000 cm^–1^ at a resolution of 2 cm^–1^, with 64 scans averaged per spectrum to improve signal-to-noise ratio.

### 2.5 Differential Scanning Calorimetry (DSC)

Differential scanning calorimetry (DSC) was performed using a DSC 25 (TA Instruments, Delaware, USA) under a nitrogen atmosphere to evaluate thermal transitions of *Spirulina*-based materials. Prior to testing, all samples were dried for 72 h in a sealed desiccator over Drierite to minimize residual moisture. Approximately 5–10 mg of material was weighed into standard aluminum DSC pans and sealed hermetically. The measurements were conducted from 25 to 250 °C at a constant heating rate of 10 °C/min. An empty sealed pan was used as reference. All data were collected in a single heating cycle. Baseline correction was applied using a spline fit excluding peak regions. Reported enthalpy values are normalized to sample mass and derived from the integrated area under each transition peak.

### 2.6 Bioinks Preparation

Bioinks were defined as the biomaterial-based inks suitable for extrusion 3D printing. Two different processing routes were used to prepare the *Spirulina*-based bioinks, as illustrated in Figure 1II. In the first method, frozen *Spirulina* was thawed at room temperature for 30 min, then transferred to an oil bath and heated to 70 °C under continuous mixing with an overhead mixer at 25 rpm. The mixture’s weight was periodically monitored until a target liquid phase content, the remaining growth medium, of 57 wt % was reached (Figure S1). HEC was manually incorporated at 5, 10, and 15 wt % relative to the biomass weight. In the second method, frozen *Spirulina* was freeze-dried (Labconco) for 72 h. The resulting dry biomass was ground into a fine powder using a mortar and pestle and then mixed with an aqueous solution of HEC predispersed in deionized water to reach the same liquid content (57 wt %) and HEC concentrations (5, 10, and 15 wt % relative to biomass weight) as in the heated and mixed formulation.

**Figure 1:**
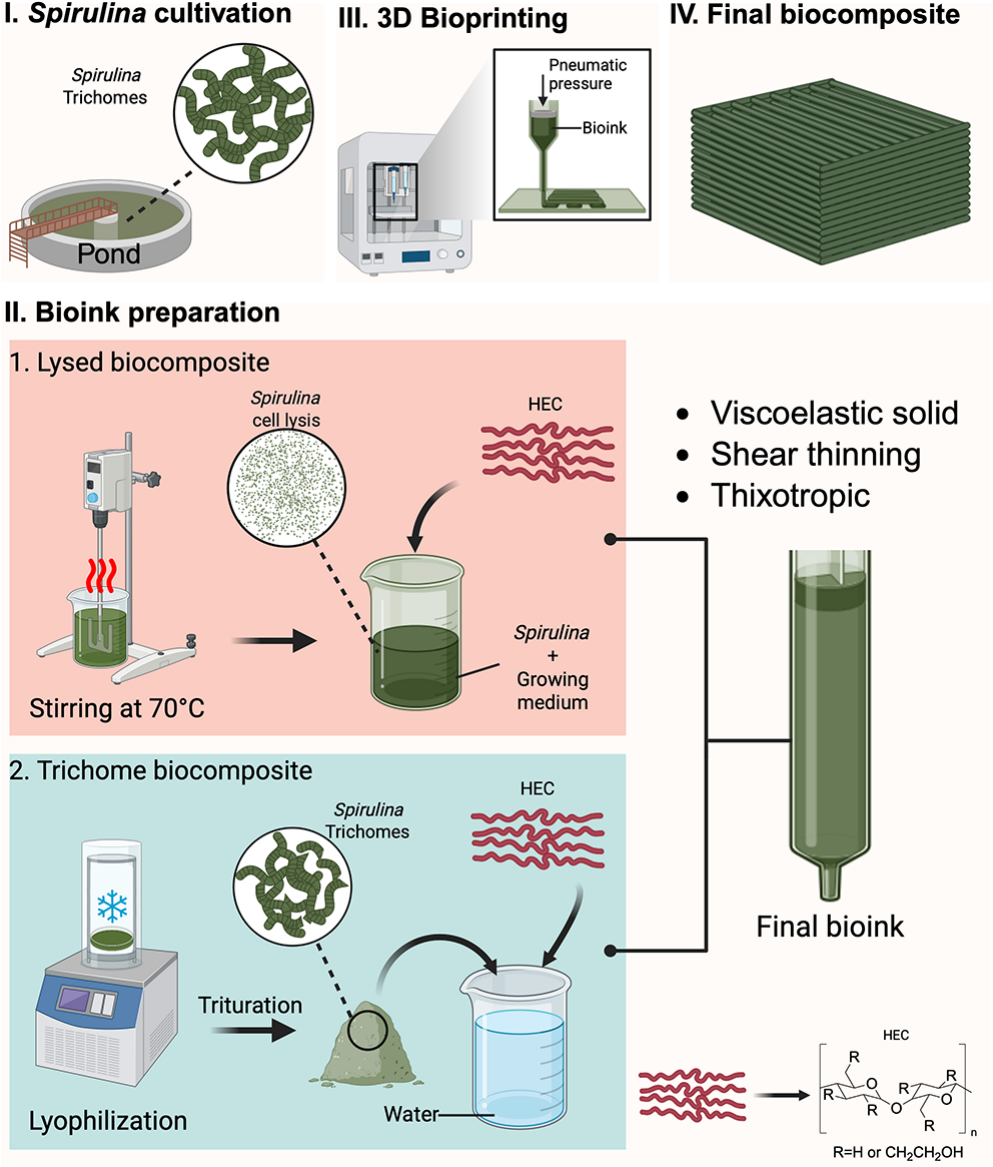
Schematic diagram of the processing methods used to produce *Spirulina* trichome-based biocomposites and lysed cell biocomposites. *Spirulina* was cultivated from a pond in growth media (I) before being prepared into a bioink via two methods (II): (1) stirring and heating, leading to cell lysis and the breakdown of the *Spirulina* trichomes, and (2) lyophilization and trituration, producing a powder which was rehydrated with water. The resulting bioinks were combined with hydroxyethyl cellulose (HEC) to produce the final bioink, which was 3D printed into final geometries (III–IV).

### 2.7 Rheological Characterization of Bioinks

The rheological properties of the different bioinks for extrusion 3D printing were measured using a dynamic rheometer (Discover HR-20 hybrid rheometer, TA Instruments, Delaware, USA). The tests were performed using a gap of 1 mm and a solvent trap to prevent liquid evaporation using a 40 mm diameter parallel plate geometry. All tests were completed at 20 °C. Flow tests were conducted to evaluate the shear thinning behavior of the bioinks at shear rates (γ̇) ranging from 10^–2^ to 10^2^ s^–1^. To identify the linear viscoelastic regime (LVR) of the bioinks, oscillatory amplitude sweep tests were conducted over a stress range of 10^–3^ to 10 kPa at a constant frequency of 1 Hz. The yield stress (τ_*y*_) for each bioink was determined as the stress at which the storage modulus (G′) intersected the loss modulus (G′′). For the viscosity (η) recovery test, the viscosity of each bioink was first measured at a shear rate of 0 s^–1^ for 60 s, then increased to 200 s^–1^ for 10 s, and finally returned to 0 s^–1^ for an additional 60 s.

### 2.8 3D Printing of Materials

Bioinks were loaded into 10 mL disposable plastic syringes and centrifuged for 2 min at 3000 rpm (5804, Eppendorf) to remove bubbles and pack the material to the bottom of the syringe. The rubber cap of the syringe plunger was placed on top of the material before printing. Samples were printed using an Allevi 2 Bioprinter (3D Systems, PA, USA) with a 14-gauge (1.6 mm) nozzle into structures measuring 15 × 15 × 7 mm^3^, with a 1.4 mm layer height. Printing speed and pneumatic pressure were adjusted to match the mass-flow rate of each bioink to ensure constant sample mass across formulations, compensating for viscosity differences (Table S1).

Samples were printed directly onto a porous and nonstick 0.003” thick fiberglass sheet coated with polytetrafluorethylene (McMaster-Carr, IL, USA). Before mechanical testing, the samples were dried. The drying process of the samples was controlled to prevent variability and cracking that could be introduced as water evaporated. After printing, the samples were weighed and then transferred to square Petri dishes (surface area 500 cm^2^) containing a 3 mL Petri dish of dried silica gel. Silica gel was replaced, and samples weighed every 24 h until constant mass was achieved, indicating complete drying. To allow a gradual drying process, until 50% of the water present in the samples had evaporated, the mass of silica gel added matched the total sample weight. After this point, twice the sample weight in silica gel was used. Final volume was calculated using averaged measurements of length, width, and height, and used to determine volumetric shrinkage and density.

### 2.9 Mechanical Analysis

Quasi-static uniaxial compression tests were conducted to evaluate the effect of cell morphology and HEC concentration on the mechanical properties of *Spirulina*-based materials. At least five cubic samples (∼11 × ∼11 × ∼4 mm^3^) were tested using an Instron E3000 universal testing machine (Instron, MA, USA) equipped with a 5 kN loadcell, at a constant strain rate of 0.001 s^–1^. Prior to testing, all samples were polished to ensure flat and parallel surfaces to minimize contact artifacts. From the measured force (F) and displacement (d) data, we calculate the stress, σ from σ = F/A, where A is the cross-sectional area, and strain from ε=d/H, where H is the initial sample height. From the stress–strain plots, the specific compressive modulus (E/ρ), the specific compressive strength (yield strength/ρ), and the maximum stress at the end of the experiment (strength) were calculated. The specific modulus was calculated from the initial linear elastic part of the compression experiments and normalized to the density of each respective sample. The yield strength was calculated using the 0.2% offset yield strength method on the stress–strain curve. The compressive strength was determined by the stress at the final point of the compression experiment, which was stopped when the samples were at ∼60% strain (as the samples did not reach total failure). The mechanical data was processed and analyzed using Python (version 3.12.7).

### 2.10 Scanning Electron Microscopy (SEM)

The structural analysis of specimens following three-point bending test failure involved surface staining with OsO_4_. An aqueous solution of 4% OsO_4_ was evaporated over the region of interest at room temperature for 1 h. High-resolution scanning electron microscopy (HRSEM) was the used to examine the micromorphology of the 3D-printed material. Images were taken using a Nova600 NanoLab system (Thermo Fisher Scientific) equipped with a secondary electron (SE2) detector, at a working distance of 5 mm and an acceleration voltage of 5 kV. Prior to imaging, samples were coated with a 5 nm platinum layer using a high-resolution sputter coater (Cressington 208HR).

### 2.11 Statistical Analysis

Quantitative results are expressed as mean ± standard deviation (SD). All statistical analyses were conducted using Python (version 3.12.7) with the statsmodels and pandas libraries. Two-way analysis of variance (ANOVA) was applied to determine whether significant differences existed between the mean values of the experimental groups. First, a linear model was specified using ordinary least-squares (OLS) with the ols­() function, incorporating both main effects and their interaction term. The resulting model was passed to anova_lm­() to generate a Type II ANOVA table. A difference between the groups was considered to be statistically significant at *p* < 0.05.

## 3 Results and Discussion

This study examines how the cellular microstructure of *Spirulina platensis* and the incorporation of hydroxyethyl cellulose (HEC) influence cohesion, printability, and mechanical behavior in sustainable algae-based materials. Specifically, we investigate how cellular architecture (maintained in trichomes or disrupted by cell lysis) affects structure–property relationships, and how HEC contributes to cohesion in either trichome-based or lysed cell formulations. *Spirulina* was cultivated in open ponds and processed via two distinct routes to prepare printable bioinks (Figure 1I–II). In the first, thermal treatment and homogenization induced complete cell lysis, eliminating trichome morphology and releasing intracellular contents. The resulting paste was combined with HEC to form a homogeneous biocomposite (Figure 1II-1). In the second route, freeze-dried *Spirulina* was rehydrated to retain trichome morphology and cell wall integrity. In this case, the *Spirulina* trichomes served as structural elements within the HEC matrix (Figure 1II-2). Varying concentrations of HEC were incorporated to evaluate its role as a binder. All formulations were extrusion-printed into small bricks at room temperature (Figure 1III–IV), dried, and mechanically tested under uniaxial compression. Rheological characterization showed that both trichome- and lysed-based bioinks behaved as viscoelastic solids with shear-thinning and thixotropic properties, enabling controlled extrusion and structural stability during printing. This approach enables direct assessment of how microstructural preservation and matrix interactions govern the performance of *Spirulina*-based materials.

### 3.1 Cell Lysis Disrupts Trichome Morphology and Structural Continuity

To evaluate the effect of processing on *Spirulina* cellular architecture, confocal microscopy was used to visualize microstructural integrity across conditions. As a reference, freshly frozen *Spirulina* biomass exhibited the characteristic helical trichome morphology, with tightly coiled multicellular filaments clearly visible and exhibiting strong autofluorescence (Figure 2A). This structure reflects the native organization of the biomass prior to any drying or thermal treatment. Following thermal treatment, *Spirulina* underwent extensive cell lysis, resulting in the complete loss of trichome architecture. Confocal imaging showed a homogeneous distribution of intracellular contents with no remaining filamentous structures (Figure 2B), indicating full rupture of the cell wall and membrane systems. This observation is consistent with previous reports identifying *Spirulina* cell lysis by optical microscopy through cell wall rupture and dissociation into subcellular fragments.^21^ In contrast, freeze-dried and powdered *Spirulina* retained the presence of cell walls and elements of trichome morphology (Figure 2C), although some fragmentation and loss of uniform coils from the initial structure was observed. Filaments remained largely structurally intact and visibly entangled compared to the lysis observed after heating and mixing, forming a network of helically coiled strands. These observations confirm that freeze-drying is a sufficient method for maintaining the physical structure of *Spirulina* for purposes of assessing the effect of morphological differences resulting from the two processing methods, including the microscale physical interactions such as interlocking that contribute to mechanical cohesion in the resulting material.

**Figure 2:**
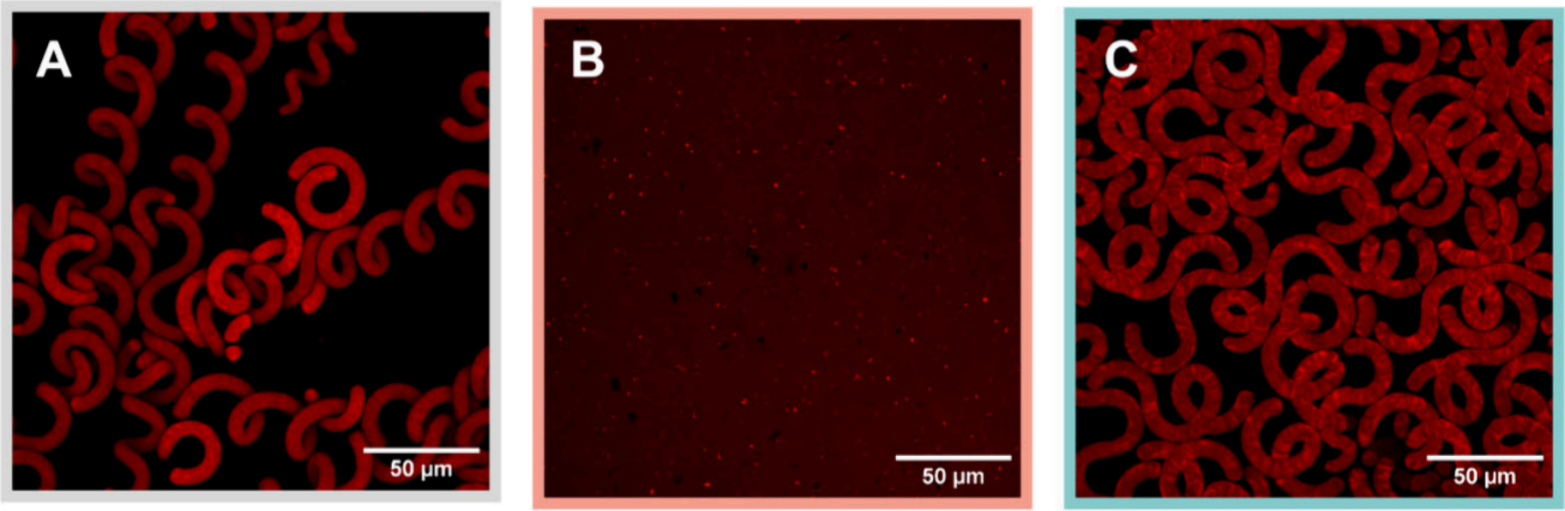
Microstructure of the *Spirulina* cells in its native state (A) is destroyed after cell lysis in producing the bioink to print a lysed biocomposite material (B), but largely maintained in overall filament structure when lyophilized and crushed into a powder used to create the bioink to print a trichome biocomposite (C).

### 3.2 Impact of *Spirulina* Microstructure on Thermal Behavior and Cohesion

Intermolecular interactions between *Spirulina* biomass and HEC play a central role in determining the cohesive strength and structural integrity of the resulting material. These interactions influence not only the internal organization of the biocomposites, but also its macroscopic mechanical behavior, including stiffness and strength, and overall integrity under load. In systems where cellular architecture is disrupted by lysis, the exposed biopolymers may engage more freely with the HEC matrix, potentially enhancing molecular-level cohesion but compromising structural reinforcement. In contrast, trichomes contribute to mechanical performance through physical interactions (ie interlocking of filaments), structural integrity of the cell wall, and spatial confinement, which together may limit molecular mixing with the matrix but can enhance bulk cohesion and mechanical integrity. Understanding the nature of intermolecular interactions is essential for elucidating the structure–property relationships that govern cohesion, load transfer, and mechanical performance in these material systems.

To elucidate the cohesive interactions between *Spirulina* and HEC, we initially performed attenuated total reflectance-infrared spectroscopy (ATR-FTIR) to assess compositional and interfacial differences. As shown in Figure S2, only minor spectral changes were observed following HEC addition, including reduced band intensities and minor enhancements in C–O (∼1000–1150 cm^–1^) and amide-associated (∼1600–1700 cm^–1^) regions. Thermal treatment to 70 °C as well as lyophilization are likely to have induced molecular-level changes to the materials. However, due to the overlapping nature of biopolymer signals and the limited resolution of these spectra for detecting weak intermolecular interactions in complex biopolymer matrices, the results were inconclusive. Consequently, we turned to differential scanning calorimetry (DSC) for a more sensitive assessment of thermal transitions associated with molecular organization and intramolecular interactions.

DSC enabled evaluation of both the intrinsic thermal behavior of *Spirulina* (intact vs lysed) and the impact of HEC incorporation (Figure S3). Lysed *Spirulina* exhibited four endothermic transitions at 66, 109, and 165 °C, with respective enthalpy values of 0.67, 0.97, and 1.12 cal/g (Figure S3A). The sharp peak at 66 °C is attributed to the loss of bound water associated with exposed hydrophilic domains following cell disruption.^22^ The broader and lower-enthalpy transition at 109 °C corresponds to protein denaturation, suggesting less defined tertiary structures in the lysed state. The prominent peak at 165 °C likely reflects relaxation or reordering of disordered polysaccharide domains, now unconstrained by cellular structure. Degradation was observed at 207 °C.

Trichome samples exhibited comparable transitions at 52, 113, and 161 °C (Figure S3B). The 52 °C moisture-related peak was broader and lower in enthalpy (0.63 cal/g), suggesting more heterogeneous water retention in the intact multicellular structure. The 113 and 161 °C transitions, attributed to protein unfolding and polysaccharide rearrangement, respectively, had higher enthalpies (1.37 and 0.97 cal/g) than their lysed counterparts, indicating greater thermal stability within the retained architecture. The degradation peak at 210 °C was significantly smaller than in lysed samples, supporting the hypothesis that structural arrangement of the biocomponents within the cell wall suppresses bulk thermal decomposition.

The biocomposite retained much of the thermal signature of unmodified trichomes (Figure S3B). Endothermic transitions at 65, 84, 160, and 207 °C were observed, with enthalpy values of 0.33, 0.95, and 0.84 cal/g for the first three peaks. The sharp polysaccharide and degradation transitions indicate that the structural integrity of trichomes is largely maintained. The 84 °C transition, absent in the trichome control, may reflect localized HEC interaction with exposed biomolecular surfaces, though the overall thermal response suggests limited molecular integration.

When HEC was added to lysed *Spirulina* (Figure S3A), the thermal profile changed substantially. A broad transition appeared at 148 °C with an enthalpy of 4.8 cal/g. The emergence of this high-enthalpy peak suggests strong thermal interaction between HEC and the lysed matrix, consistent with molecular-level mixing. The absence of a distinct degradation peak and the redistribution of thermal energy imply that HEC alters the supramolecular organization of the lysed biocomposite by interacting with exposed polysaccharide and protein residues.

Taken together, these results demonstrate that cell lysis facilitates strong cohesive interactions with HEC, as evidenced by broader transitions and higher enthalpy in the lysed biocomposite. In contrast, trichomes resist such mixing, maintaining physical cohesion and well-defined thermal domains.

### 3.3 Rheological Behavior of *Spirulina*-Based Bioinks

Rheological analysis was performed to elucidate how *Spirulina* microstructure and HEC concentration influence viscoelastic behavior, flow response, and structural recovery, all of which are key properties governing printability and shape fidelity in extrusion-based 3D printing (Figure 3). Understanding how cellular architecture (intact trichomes vs lysed biomass) and binder content affect these parameters is essential for optimizing formulation and material processing.

**Figure 3:**
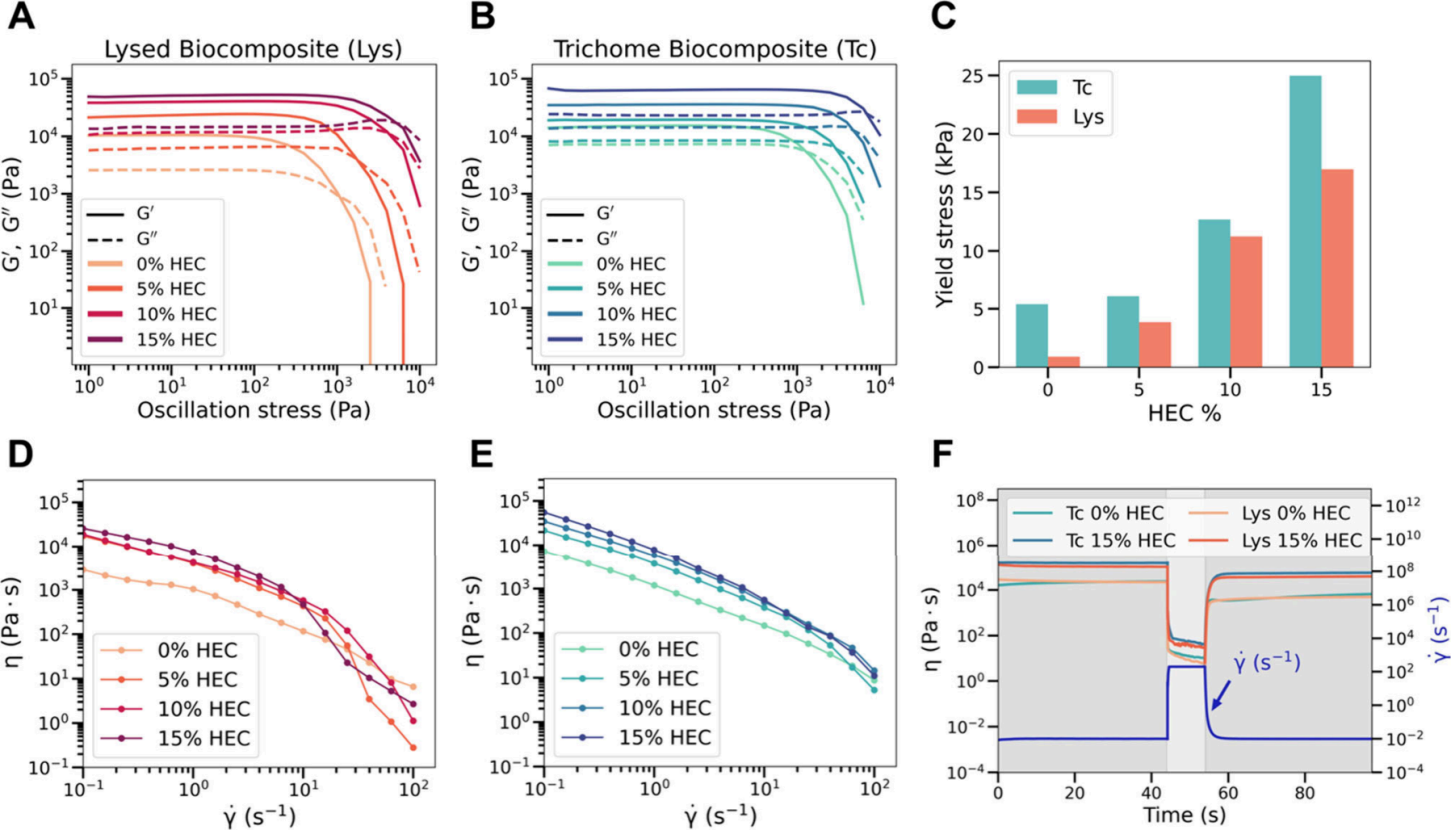
(A–C) Rheological properties of *Spirulina* in a lysed biocomposite (Lys) and trichome biocomposite (Tc). (D, E) All *Spirulina*-HEC bioinks tested demonstrate shear thinning behavior. (F) Viscosity recovery and shape retention of *Spirulina*-HEC bioinks.

All formulations behaved as viscoelastic solids, exhibiting G′ > G′′, and displayed a yield stress defined by the crossover point of G′ and G′′ under oscillatory stress (Figure 3A–C). Trichome-based bioinks consistently showed higher G′ and G′′ values at low stress across all HEC concentrations, indicating stronger elastic and viscous responses. For HEC-free formulations, the yield stress of trichome suspensions reached ∼5 kPa, compared to ∼2 kPa for lysed-cell suspensions (Figure 3C). One possible explanation for this difference is increased physical interactions and interlocking between trichomes and the structural rigidity of intact cell walls, which enable formation of an elastic network that resists deformation. In contrast, the lysed biomass formed a more homogeneous suspension of smaller particulates that flowed more readily, resulting in lower yield stress. As HEC content increased to 15 wt %, yield stress values rose to ∼25 kPa for trichome biocomposites and ∼15 kPa for lysed biocomposites, suggesting that trichomes enhance matrix cohesion even when spatially confined due to structural trichome interactions and physical interlocking. These trends were reflected in the printing speeds and pneumatic pressures required to achieve a consistent printed sample mass across different bioinks. Bioinks of the trichome biocomposites required a higher printing pressure than the lysed biocomposites by up to 26.6 PSI. In addition to the printing speed being decreased by 2 mm/s for each 5 wt % increase in HEC, bioinks of higher HEC content required a greater printing pressure. Specifically, the pressure increased by 41.0 PSI in the trichome biocomposite and 43.2 PSI in the lysed biocomposite when comparing 5% to 15% HEC content (Table S1).

Flow behavior measurements showed that all bioinks exhibited shear-thinning behavior (Figure 3D–E). Trichome-based formulations consistently demonstrated higher viscosities than their lysed counterparts across all shear rates, reflecting enhanced flow resistance, potentially due to filament entanglement and physical interactions of larger multicellular particulates. The presence or concentration of HEC did not substantially alter the shear-thinning profile, though higher HEC levels generally increased viscosity due to its hygroscopic nature and gel-forming behavior, which increases flow resistance.^21^ Notably, viscosity profiles of trichome-based formulations were more linear, suggesting greater network stability and reduced shear sensitivity, likely resulting from persistent filamentary interactions and cell wall integrity. These features contributed to more stable extrusion behavior during printing. Thixotropic recovery tests confirmed that all bioinks partially regained viscosity following shear disruption (Figure 3F). Although full recovery was not observed, the rapid rebound was sufficient to maintain printed shape fidelity, supporting the suitability of these formulations for extrusion-based processing.

### 3.4 3D-Printed Structure Characterization

Mechanical strength and stiffness are essential for materials intended for structural applications, including sustainable packaging, construction panels, and load-bearing components. These properties are governed not only by composition but also by internal architecture and mechanisms of stress transfer across the material.^34^ In particular, the preservation or disruption of *Spirulina*’s cellular structure may influence how stress is transmitted, absorbed, and resisted within the matrix. To investigate this, we examined how differences in microstructure (trichomes vs lysed cells) and HEC concentration affect bulk mechanical behavior. We conducted quasi-static uniaxial compression tests on dried, 3D-printed samples to assess the stiffness, yield strength, and strength of trichome-based biocomposite and lysed cell biocomposite formulations (Figure 4 and Table 1). While this work focused on uniaxial loading under compression, future work could potentially examine more complex loading scenarios such as bending and tension. Formulations prepared without HEC (0 wt %) exhibited extensive cracking and structural failure during dehydration, which prevented mechanical testing. This observation underscores the role of HEC as a binder in maintaining cohesion and dimensional stability in algae-based materials.

**Figure 4:**
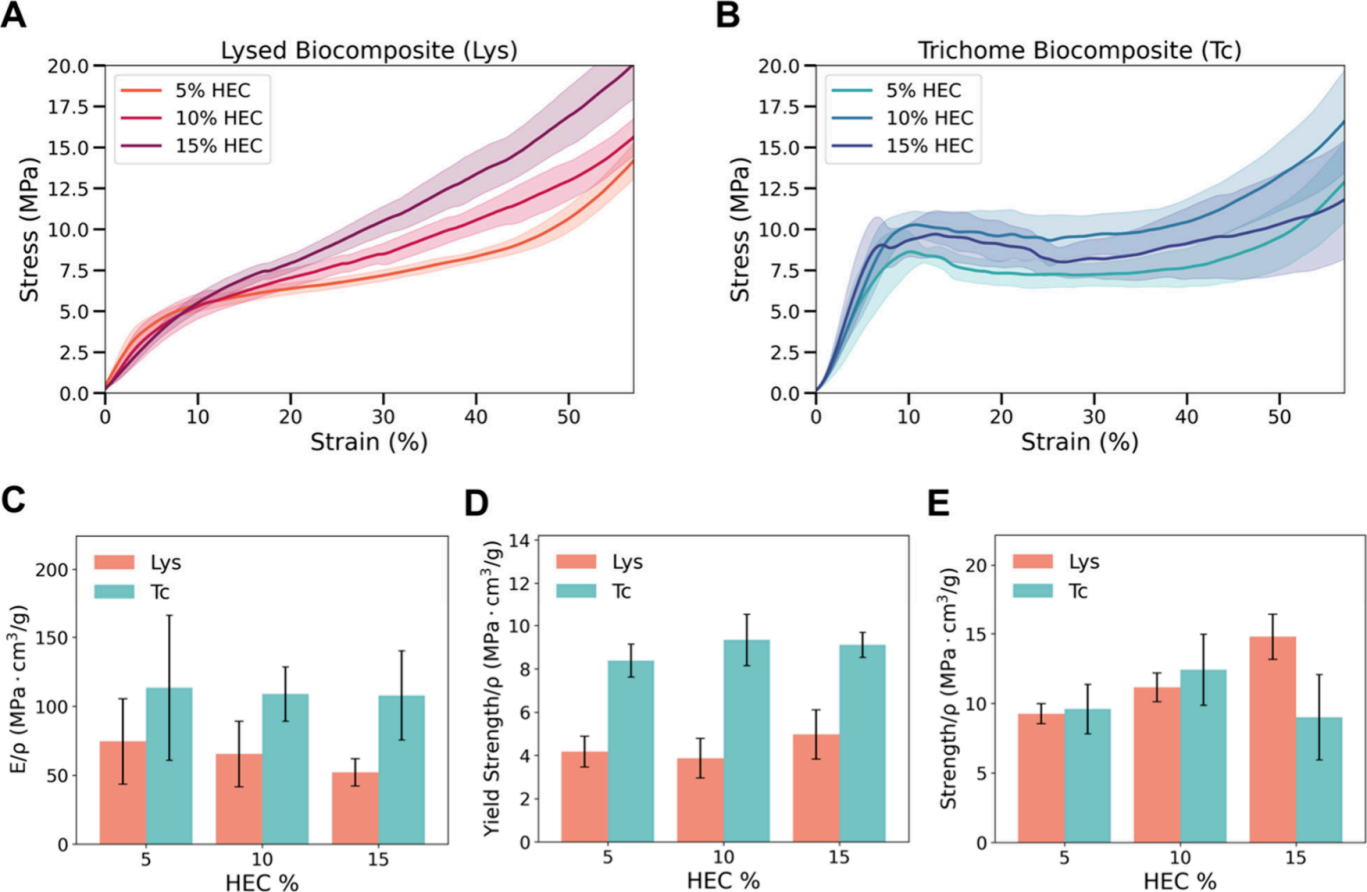
(A, B) Stress–strain curves from compression tests of *Spirulina*-HEC biocomposites from lysed biomass or trichomes. (C–E) The normalized modulus, yield strength, and strength of the samples calculated from compression tests.

**Table 1:** Mechanical Properties of *Spirulina*-HEC Biocomposites

	HEC %	E/ρ (MPa·cm^3^/g)	Yield strength/ρ (MPa·cm^3^/g)	Strength/ρ (MPa·cm^3^/g)
Trichome biocomposite	5	113.41 ± 52.65	8.38 ± 0.76	9.59 ± 1.77
	10	108.90 ± 19.71	9.34 ± 1.19	12.41 ± 2.56
	15	107.81 ± 32.33	9.11 ± 0.58	9.00 ± 3.08
Lysed biocomposite	5	74.54 ± 30.98	4.81 ± 0.78	9.25 ± 0.72
	10	65.35 ± 23.56	4.49 ± 0.89	11.15 ± 1.03
	15	52.03 ± 9.97	5.69 ± 1.30	14.78 ± 1.62

Volumetric shrinkage and bulk density measurements (Figure S4, Table S2) further reflect the impact of *Spirulina* microstructure and HEC content on drying behavior and material consolidation. Trichome-based formulations exhibited consistently lower shrinkage values (58–62%) than those with lysed biomass (67–69%), indicating enhanced resistance to collapse during dehydration. A two-way ANOVA confirmed that both microstructure (*p* = 2.46 × 10^–4^) and HEC concentration (*p* = 1.32 × 10^–2^) significantly influence shrinkage (Table S3d). Density trends mirrored these differences: lysed biocomposites maintained relatively high and stable densities (1.14–1.17 g/cm^3^), while trichome biocomposites showed lower values at 5–10 wt % HEC (1.00–1.08 g/cm^3^), increasing to 1.13 g/cm^3^ at 15 wt %. Statistical analysis again showed significant effects of both formulation (*p* = 2.17 × 10^–8^) and HEC level (*p* = 3.27 × 10^–4^) on density (Table S3e). These results suggest that intact trichomes help retain open structure and reduce compaction during drying, while HEC modulates water retention and packing efficiency, influencing the final material properties. This trend further supports the hypothesis that the spiral morphology of trichomes contributes to maintaining the internal architecture of the material, limiting volumetric collapse and promoting structural stability throughout dehydration.

The internal structural integrity conferred by the cellular structure was also reflected in the mechanical behavior of each material. Average stress–strain curves for both lysed biocomposites (Figure 4A) and trichome biocomposites (Figure 4B) revealed distinct deformation profiles. Lysed biocomposites exhibited a typical response of homogeneous, particle-based systems, with an initial linear elastic regime followed by yielding and moderate strain hardening.^33^ In contrast, trichome biocomposites displayed an initial linear region followed by a pronounced plateau before densification, consistent across HEC concentrations. This plateau likely corresponds to the progressive collapse or rearrangement of trichomes within the matrix. Additionally, physical interlocking between trichomes may restrict their relative motion under compression, contributing to a more gradual stress response and enhanced energy dissipation. The observed high variability of the strength-strain curves observed in both biocomposites is characteristic of biological materials due to inherent heterogeneity within the material and the presence of defects,^35^ which are likely introduced during sample fabrication and drying.

The differences in microstructure were also reflected in the normalized stiffness and yield strength of the printed materials (Figure 4C–D, Table 1). These trends were also present before normalization of these values to specific sample densities, which are presented in Table S4. Across all HEC concentrations, trichome biocomposites exhibited higher specific modulus than lysed biocomposites, indicating superior stiffness per unit mass. At 5 wt % HEC, the trichome biocomposite’s modulus reached 113.4 MPa·cm^3^/g, compared to 74.5 MPa·cm^3^/g in the lysed biocomposite. This trend persisted with increasing HEC content, although neither system showed a monotonic dependence on HEC concentration. A two-way ANOVA (Table S3a) confirmed that the processing method (trichomes vs lysed biomass) significantly influenced the modulus (*p* = 3.56 × 10^–5^), whereas HEC content did not (*p* = 0.148). These results suggest that the preservation of *Spirulina’s* helical trichome architecture and intact cell walls plays a dominant role in enhancing stiffness, whereas the contribution of HEC to elastic behavior is minimal.

Yield strength followed a similar pattern, with trichomes consistently outperforming lysed biomass (Figure 4D, Table 1). For example, at 10 wt % HEC, trichome biocomposites reached a specific yield strength of 9.3 MPa·cm^3^/g, compared to 4.5 MPa·cm^3^/g in the lysed biocomposites. Yield strength represents the stress required to initiate plastic deformation and is a key indicator of a material’s load-bearing capacity. Statistical analysis (Table S3b) again confirmed a highly significant effect of processing method (*p* = 4.10 × 10^–10^), but no significant effect of HEC concentration (*p* = 0.925). The improved performance of the trichomes biocomposites reflects the mechanical stability imparted by the structural continuity and interlocking of *Spirulina* filaments within the HEC matrix. In contrast, the more homogeneous and isotropic structure of the lysed biomass (composed of smaller, fragmented particulates) limits stress transfer and structural resistance under compression.

The normalized compressive strength, defined as the maximum stress during testing, exhibited distinct trends across formulations (Figure 4E, Table 1). Strength was significantly influenced by both HEC concentration (*p* = 5.00 × 10^–5^) and processing method (*p* = 1.14 × 10^–3^, Table S3c). Lysed biocomposites showed a continuous increase in strength with HEC content, peaking at 14.78 MPa·cm^3^/g at 15 wt %, likely due to densification and enhanced cohesion among lysed cell particulates. In contrast, trichome biocomposites reached maximum strength (12.41 MPa·cm^3^/g) at 10 wt % HEC but declined at 15 wt %, suggesting that excessive binder may disrupt the reinforcing network formed by trichomes. We suggest that while trichome architecture governs stiffness and yield behavior, compressive strength in lysed biocomposites is more sensitive to matrix compaction and binder-driven cohesion.

SEM imaging of fractured cross sections revealed that microscale cracking occurred across all sample types, both in the absence and presence of HEC, regardless of processing method (trichomes or lysed biomass) (Figure S5). While the inclusion of HEC was essential for preventing large-scale collapse during dehydration, the presence of persistent internal cracks suggests that HEC alone did not provide sufficient mechanical reinforcement at the microscale to fully maintain cohesion upon drying. To overcome this structural limitation, we suggest that additional strategies, such as alternative dehydration protocols or incorporation of secondary binders, may be necessary to minimize internal damage and enhance structural integrity in dried *Spirulina*-based materials.

In context of previous work investigating whole *Spirulina* biomass biocomposites, the effect of *Spirulina* microstructure on biocomposite mechanics depends strongly on matrix chemistry and processing. Improved mechanical properties in *Spirulina*–PLA composites, following cell disruption, have been attributed to increased particulate surface area and stress transfer in a melt-processed matrix (∼170 °C) in a primarily PLA matrix.^32^ In contrast, our biocomposites contain higher biomass fractions (up to ∼85 wt %) and are processed at room temperature with minimal relative amounts of HEC binder, where the *Spirulina* biomass dominates the structure. Retaining filamentous trichome morphology in this context enables potential mechanical interlocking without relying on thermal processing or strong polymer–filler interactions, isolating the structural function of the *Spirulina* cell wall and cellular architecture itself. Jiang et al.^36^ similarly showed ball milling enhanced mechanical performance by facilitating covalent bonding between *Spirulina* proteins and a polyimine network, emphasizing that cell disruption is beneficial when matrix formation depends on chemical reactions. Taken together, these results indicate that the mechanical advantage of maintaining or disrupting *Spirulina* microstructure is not universal, but rather dictated by the composite design strategy. While in thermally or chemically driven composite systems, cell disruption may be advantageous, in low-energy, room-temperature systems such as those demonstrated in the present work, preserving native multicellular architecture in gentler processing methods can confer stronger mechanical properties.

## 4 Conclusion

This study demonstrates that preserving the native microstructure of *Spirulina platensis* enhances the performance of biobased materials produced by extrusion 3D printing. By comparing formulations made from freeze-dried trichomes and thermally lysed cells, we show that cellular architecture plays a decisive role in rheological behavior, drying response, and mechanical performance. Freeze-dried *Spirulina* retained its filamentous morphology, leading to higher ink viscosity and yield stress potentially due to interlocking of trichome architecture. In contrast, thermal treatment disrupted the helical structure and cell walls, producing more flowable biomass with lower cohesion. Although lysed cells exhibited stronger molecular interactions with HEC, trichomes offered superior mechanical reinforcement through physical interlocking, preservation of the cell wall, and improved stress transfer. Trichome-based biocomposites achieved a significantly higher specific modulus and yield strength, as well as reduced volumetric shrinkage during drying. Hydroxyethyl cellulose was essential to prevent macroscopic cracking, but SEM revealed persistent microscale fractures in all samples, indicating limits in internal cohesion at the tested binder levels. These results highlight the importance of gentle processing routes to retain biological structure and maximize material functionality. Future work should aim to reduce cracking and improve mechanical robustness by exploring alternative binders, fiber reinforcement, or optimized drying protocols, guiding the development of *Spirulina*-based materials as viable, sustainable alternatives for structural applications.

## Supplementary Material



## Data Availability

The data that support the findings of this research are available from the corresponding author upon reasonable request.

## Notes

TGA thermograms of heated and mixed lysed *Spirulina* bioink and powdered trichome *Spirulina*; ATR-FTIR spectra; DSC spectra; printing parameters used; volumetric shrinkage and density data; scanning electron microscopy images; photographs of representative samples; ANOVA results; non-normalized mechanical data values (PDF)

## §

Department of Materials Engineering, Ben Gurion University of the Negev, 8410501 Beer-Sheva, Israel
