# Supplementary material for: Preserving Microstructure Enhances Cohesion and Mechanical Performance in Spirulina-Based 3D-Printed Biomaterials
Source: ACS Appl Eng Mater. 2026 Jan 16;4(2):963–71. doi: 10.1021/acsaenm.5c01105 (PMC12954744; doi:10.1021/acsaenm.5c01105)
Supplement: Supplementary file 1 [file em5c01105_si_001.pdf]

Supporting Information

Preserving Microstructure Enhances Cohesion and Mechanical Performance in *Spirulina*-Based 3D-Printed Biomaterials

Amelia Burns[a], Israel Kellersztein[b,*,1], and Chiara Daraio[b,*].

[a]Division of Biology and Biological Engineering, California Institute of Technology, 1200 E California Blvd, Pasadena, CA, USA.
[b]Division of Engineering and Applied Science, California Institute of Technology, 1200 E California Blvd, Pasadena, CA, USA.

[1]Current address: Department of Materials Engineering, Ben Gurion University of the Negev, Beer-Sheva, Israel.

*Corresponding authors:
Israel Kellersztein: israelke@bgu.ac.il
Chiara Daraio: daraio@caltech.edu

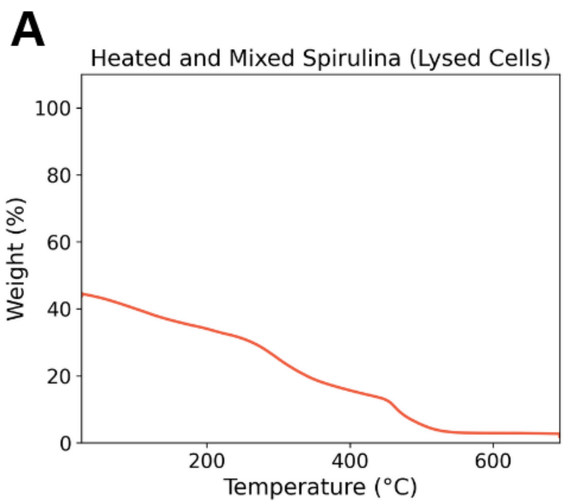

Fig. S1: Thermogravimetric analysis of A) heated and mixed lysed *Spirulina* bioink and B) powdered trichome *Spirulina*. Due to the initial moisture content of heated and mixed *Spirulina*, bioink samples were set at room temperature within the instrument until reaching a stable weight before heating began. Formal start of TGA did not begin until after initial moisture had evaporated, and final weight percent was calculated according to initial total weight.

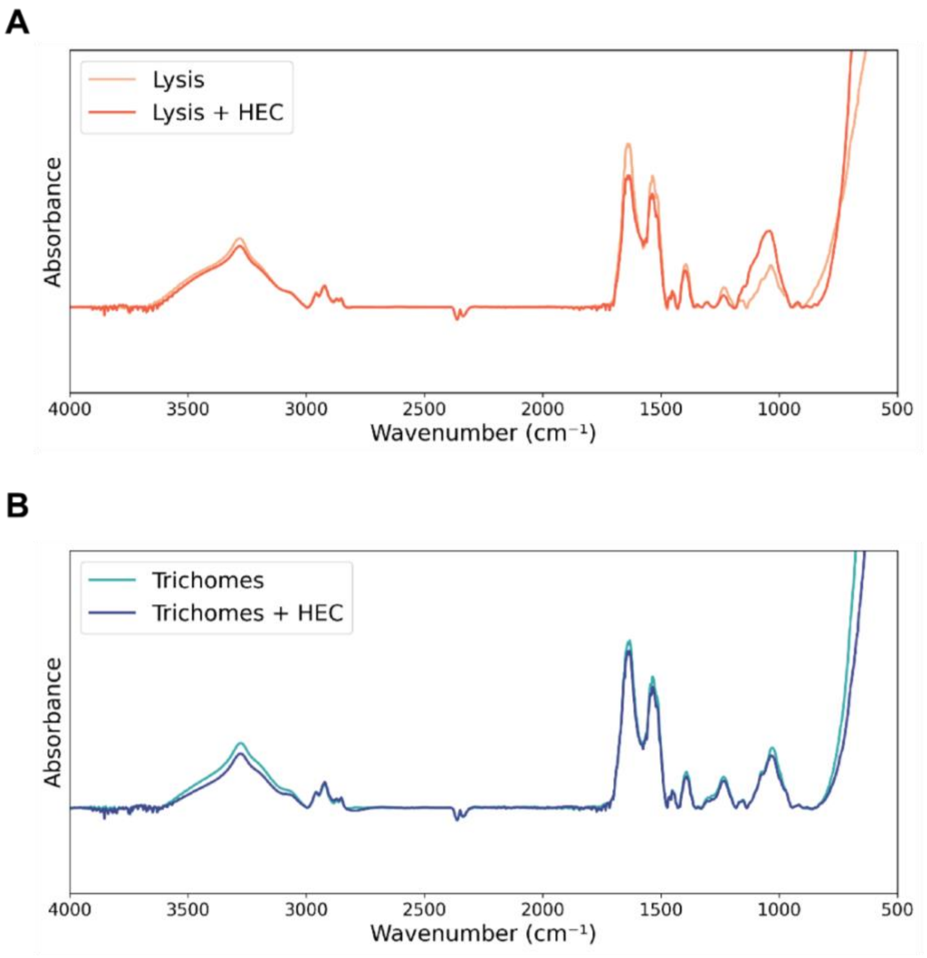

Fig. S2: ATR-FTIR spectra of A) lysed *Spirulina* cells and B) *Spirulina* trichomes.

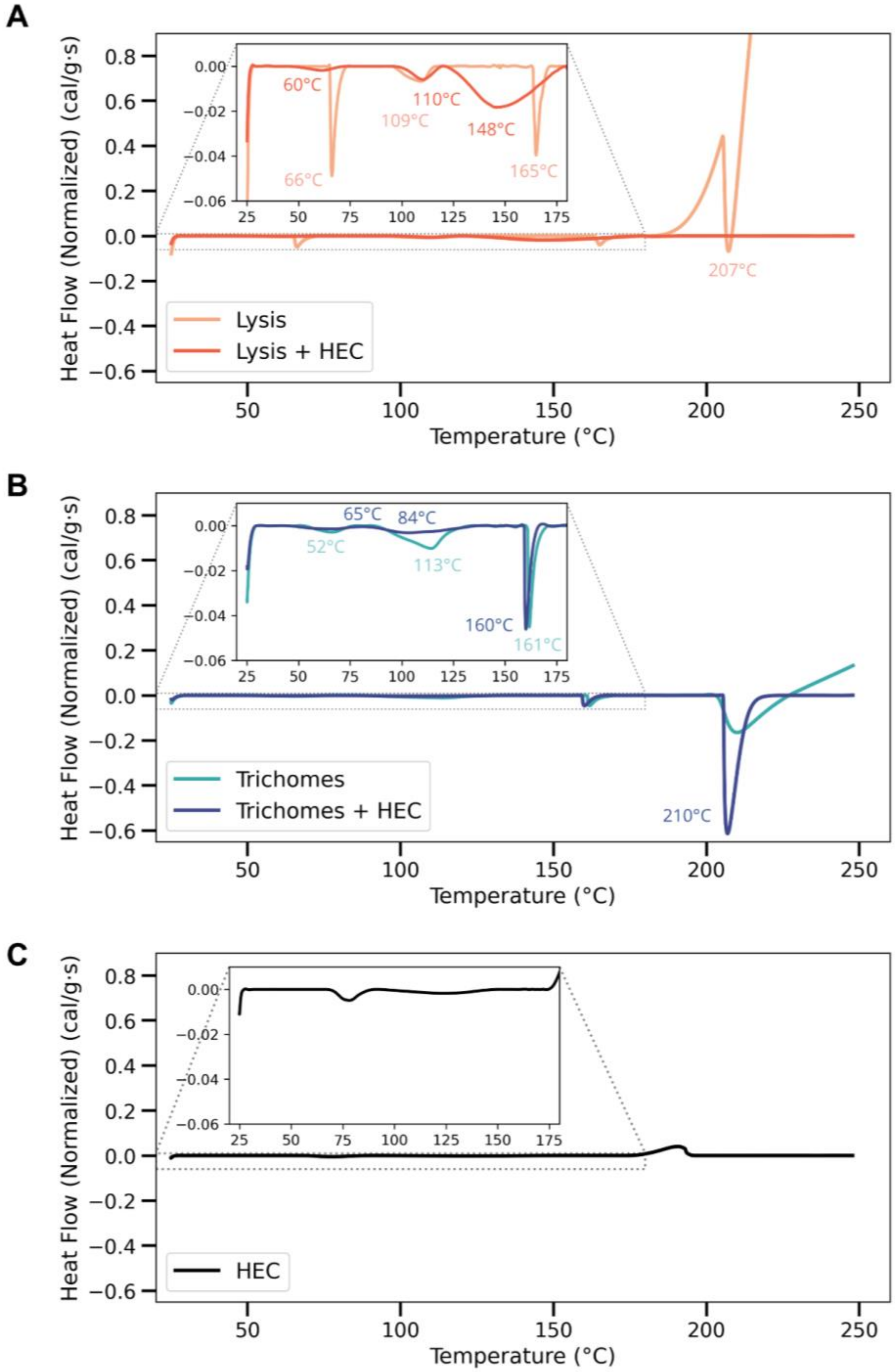

Fig. S3: Differential scanning calorimetry (DSC) spectra of A) lysed *Spirulina* cells, B) *Spirulina* trichomes, and C) hydroxyethyl cellulose (HEC).

	HEC %	Printing Pressure (PSI)	Printing Speed (mm/s)
**Trichome Composite**	**5**	38.0 ± 1.85	9
	**10**	79.0 ± 2.16	7
	**15**	79.0 ± 1.15	5
**Lysed Composite**	**5**	27.8 ± 0.27	9
	**10**	52.4 ± 1.49	7
	**15**	71.0 ± 4.86	5

Table S1: Printing parameters used for printing each bioink using an Allevi 2 Bioprinter. All samples were printed with a 14-gauge (1.6 mm) nozzle into structures measuring 15 × 15 × 7 mm^3, with a 1.4 mm layer height.

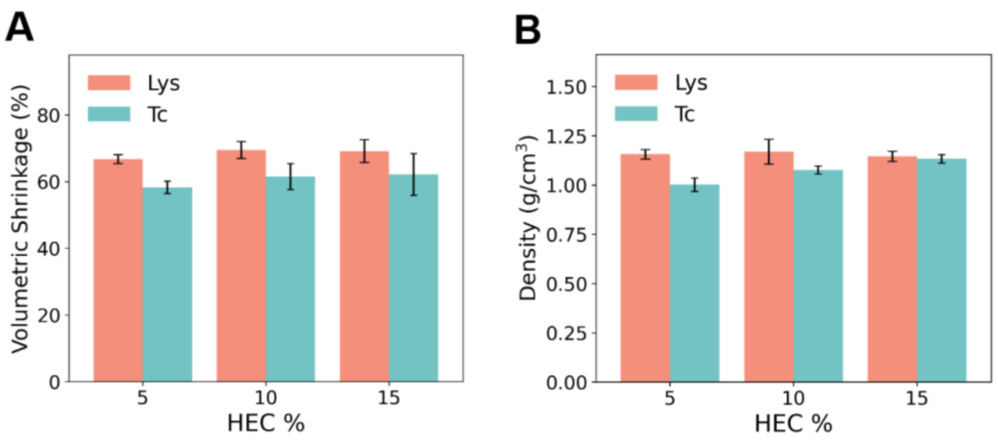

Fig. S4: A) Volumetric shrinkage and B) density of dried 3D printed *Spirulina* samples printed from each bioink.

	HEC %	Volumetric shrinkage (%)	Density (g/cm^3)
**Trichome Composite**	5	58.28 ± 1.85	1.00 ± 0.03
	10	61.52 ± 3.90	1.08 ± 0.02
	15	62.12 ± 6.27	1.13 ± 0.02
**Lysed Composite**	5	66.72 ± 1.33	1.16 ± 0.02
	10	69.46 ± 2.52	1.17 ± 0.06
	15	69.13 ± 3.43	1.14 ± 0.03

Table S2: Values of volumetric shrinkage and B) density of dried 3D printed *Spirulina* samples printed from each bioink.

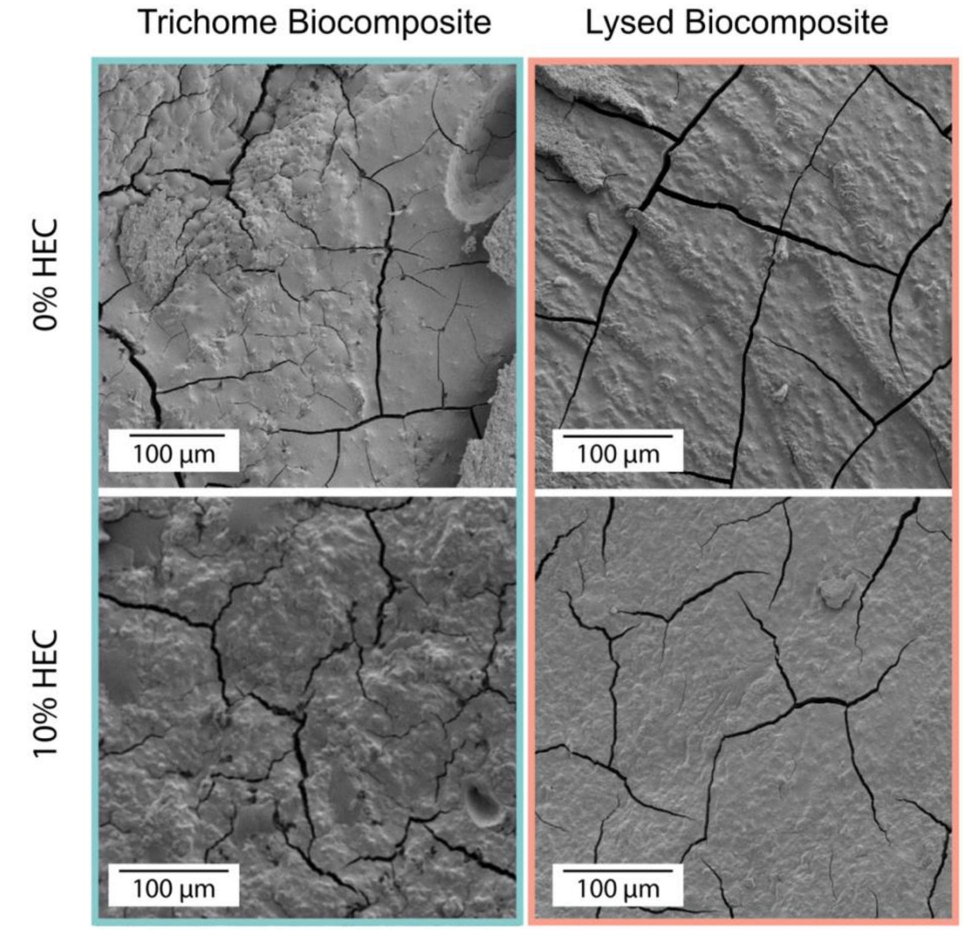

Fig. S5: Scanning Electron Microscopy (SEM) images of the internal structure of dried *Spirulina* samples with 0 and 10% HEC.

A B

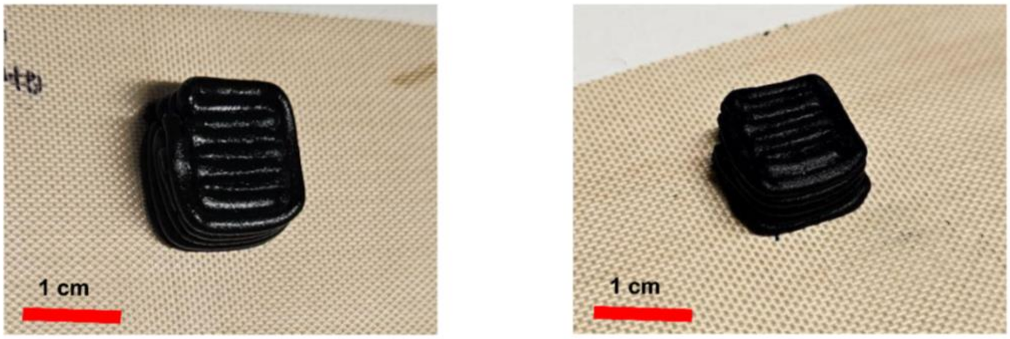

Fig. S6: Photographs of representative samples printed from (A) lysed *Spirulina* biomass composite, and (B) trichome *Spirulina* composite (each made with 10 wt% HEC).

a. E/ρ

	df	SS	MS	F	p-value
HEC %	3	4816.315	1605.44	1.90	1.48e-01
Processing Method (Tc vs Lys)	1	19082.48	19082.48	22.61	3.56e-05
Interaction	3	1846.01	615.34	0.72	5.42e-01
Residual	34	28699.22	844.09		

b. Yield Strength/ρ

	df	SS	MS	F	p-value
HEC %	3	0.72	0.24	0.15	9.25e-01
Processing Method (Tc vs Lys)	1	114.85	114.85	74.93	4.10e-10
Interaction	3	2.70	0.90	0.59	6.28e-01
Residual	34	52.11	1.53		

c. Strength/ρ

	df	SS	MS	F	p-value
HEC %	3	95.71	31.90	10.46	5.00e-05
Processing Method (Tc vs Lys)	1	38.51	38.51	12.63	1.14e-03
Interaction	3	95.00	31.67	10.39	5.30e-05
Residual	34	103.66	3.05		

d. Volumetric Shrinkage

	df	SS	MS	F	p-value
HEC %	3	204.75	68.25	4.14	1.32e-02
Processing Method (Tc vs Lys)	1	276.21	276.21	16.77	2.46e-04
Interaction	3	51.27	17.09	1.04	3.88e-01
Residual	34	560.01	16.47		

e. Density

	df	SS	MS	F	p-value
HEC %	3	0.04	0.01	8.12	3.27e-04
Processing Method (Tc vs Lys)	1	0.09	0.09	52.53	2.17e-08
Interaction	3	0.04	0.01	7.04	8.32e-04
Residual	34	0.06	0.01		

Table S3: ANOVA analysis of mechanical properties for dried *Spirulina* samples.

	HEC %	E (MPa)	Yield Strength (MPa)	Strength (MPa)
**Trichome Composite**	5	113.97 ± 52.84	8.39 ± 0.80	9.25 ± 1.91
	10	117.22 ± 22.24	10.05 ± 1.41	11.15 ± 2.96
	15	121.83 ± 35.90	10.32 ± 0.79	14.79 ± 3.52
**Lysed Composite**	5	85.99 ± 35.10	4.81 ± 0.78	9.25 ± 0.82
	10	76.58 ± 28.28	4.49 ± 0.89	11.15 ± 1.26
	15	59.50 ± 11.00	5.69 ± 1.30	14.79 ± 1.91

Table S4: Mechanical properties of *Spirulina*-HEC composites (not normalized to sample density).
